# Co-producing better land management? An ethnographic study of partnership working in the context of agricultural diffuse pollution

**DOI:** 10.1007/s41130-022-00170-6

**Published:** 2022-05-16

**Authors:** Thomas Vetter

**Affiliations:** Anthropology Institute, University of Neuchâtel, Rue de Saint-Nicolas 4, 2000 Neuchâtel, Switzerland

**Keywords:** Partnership working, Co-production, Agriculture, Diffuse pollution, Good farming

## Abstract

Partnership working has become a normative principle within agri-environmental governance. With more and more benefits becoming attributed to closer multi-stakeholder collaboration, more public monies are being directed towards this cause. These benefits have been studied widely and are usually presented in terms of their contributions to environmental, economic and/or social objectives. However, in contrast to these reported outcomes of partnership working, the practical ways towards them have received little attention. What does it mean to work together on a day-to-day basis? More specifically, how do stakeholders become trusted partners, bridge interests and coordinate their actions? What collaborative working culture becomes established within partnerships and how does this in turn affect wider governance outcomes, expectations and aspirations? Answers to these questions are not only important to better understand the factors that contribute to successful ways of partnership working, but also to account for its limitations. This paper responds to this research need by drawing on the example of Farm Herefordshire. This cross-organizational partnership promotes profitable farming, healthy soils and clean water to address the problem of diffuse pollution from agricultural practices within the Wye catchment in the UK. The insights from this case study contribute to the literature in two major ways: firstly, the paper follows prompts to study such modes of collective action holistically and bottom-up to capture all their contributions and implications. It does so by employing an ethnographic research approach to investigate the social interactions and struggles that characterize joint working. This commands attention to the backstories, the actual work meetings, the discussions, the processes of consensus building, and the joint actions undertaken; secondly, the paper connects with wider social science concerns around the underlying processes and practices of governmentality that are essential for establishing social and ecological orders. Thus, the paper explores how everyday practices of partnership working contribute to the co-production of institutions, discourses, identities, and representations—which in this case become strategically deployed to nudge—rather than revolutionise—better land management practices.

## Introduction

Several benefits—and indeed hopes—have become associated with partnership working in agri-environmental governance. These benefits are usually presented in terms of their contributions to environmental, economic and/or social objectives. Whereas collaboration between farmers, for instance, may help avoid further landscape fragmentation (Emery & Franks, 2012; Leventon et al., 2017), reduce long-term transaction costs (Franks, 2011), help (re-)build social capital (de Krom, 2017; Mills et al., 2011; Wynne-Jones, 2017), and encourage peer-to-peer learning (Lucas et al., 2019), the inclusion of non-farming actors such as environmental charity groups within wider agri-environmental collaboratives promises more balanced and legitimate outcomes over competing demands through the generation of mutual trust and understanding (Prager & Vanclay, 2010; Prager, 2015a). These latter examples of multi-actor “partnerships for sustainability governance” (McAllister & Taylor, 2015, p. 86), in particular, have come to be viewed very positively for their wide-ranging contributions to more sustainable land management. According to Prager (2015a, p. 375) those contributions can include “policy implementation and service provision; coordination and mediation; awareness raising and behaviour change; […] maintenance and protection of landscapes […]; and income generation and economic benefits.” As such partnership working is understood as a vehicle for sustainable development (Prager, 2015a, 2015b), as much as it is seen as a conduit to foster more equitable, democratic and adaptive governance systems (Hahn et al., 2006).

However, in contrast to these potential outcomes of partnership working, the actual ways towards them—including what it really means to work together on a day-to-day basis—have received little attention. So far, most studies have either approached the topic somewhat theoretically—by exploring the barriers, pathways and motivations of farmers to work together within potentially revised versions of the EU’s agri-environment schemes (Emery & Franks, 2012; Franks & Emery, 2013; Leventon et al., 2017)—or somewhat distanced, by gathering and contrasting the ex-post accounts of various actors involved in agri-environmental collaboratives (Enengel et al., 2011). More process- and practice-oriented research was rarely conducted and where this was done, the insights mainly derive from researcher-led focus group discussions (see, e.g., Leventon et al., 2017). The resulting narratives are therefore largely devoid of the social interactions and struggles that characterize partnership working, and most studies remain silent about the dynamics that emerge from such collaborative activities. This paper aims to address this gap by exploring the everyday aspects and meanings of partnership working. In particular, how do different stakeholders become trusted partners, bridge their interests and coordinate their actions? What codes of conduct become established within partnerships? Which spaces for discussion open-up or are closed down? How are different roles and characteristics reworked within such groups? And how does all this in turn affect the representation of the governance problems and the solutions at hand?

Answers to these questions are not only important to better understand the factors that can contribute to successful ways of partnership working, but also to account for its limitations. Because, as McAllister and Taylor (2015, p. 87) pointed out “Partnerships are no panacea,” in spite of their potential benefits. As with any governance instrument, such modes of collective action entail costs and risks. They are embedded in power relations, they can become captive to certain interests, they may be lacking in transparency, accountability and inclusiveness (Dandy et al., 2014; McAllister & Taylor, 2015), and they may turn out to be counterproductive for bringing about change (Blühdorn & Deflorian, 2019; Turnhout et al., 2020).

This paper will provide more clarity about this ambivalent picture of partnership working by exploring the example of Farm Herefordshire. This cross-organizational partnership addresses the problem of diffuse pollution from agricultural practices within the Wye catchment of the UK. The insights from this case study contribute to the literature on several fronts: First, the paper follows Prager’s (2015a) prompt to study such modes of collective action holistically and bottom-up to capture all their contributions and implications. It does so by employing an ethnographic research approach to investigate the social interactions, struggles and dynamics that characterize joint working. This commands attention to the backstories, the work meetings, the discussions, the processes of consensus building, and the joint actions undertaken. Second, and most notably, the paper connects with wider social science concerns around the underlying processes and practices of governmentality, which are essential for establishing social and ecological orders (Jasanoff, 2004). Thus, the paper explores how everyday practices of partnership working contribute to the co-production of institutions, discourses, identities, and representations which in this case all become strategically deployed to nudge—rather than revolutionize—better land management practices. Finally, the paper helps to fill an empirical gap. According to Emery and Franks (2012) most insights about environmental partnership working derive from case studies in Germany, Australia, and the Netherlands, yet detailed accounts of similar examples are largely missing for the UK (but see e.g. Dandy et al., 2014; Wynne-Jones et al., 2019). Overall, the insights of this case study draw a more nuanced picture of the contributions and limitations of partnership working, which should prove useful to inform and improve similar approaches elsewhere.

The remainder of this article is structured as follows: The next section explains the theoretical perspective employed in this paper. This will be followed by the methodology (Sect. 3) and some background information about the Farm Herefordshire case study (Sect. 4). Section 5 then presents and discusses the co-products of partnership working, which will provide the basis for some concluding remarks in Sect. 6.

## The idiom of co-production and its relevance in agri-environmental contexts

Given that partnership working is increasingly common within agri-environmental contexts it is fair to say that this phenomenon is on its way to become a new normative governance principle: “With its rhetorical focus on cooperation and participation, partnerships seem to embody the inclusive, joint problem-solving approaches promoted in sustainability discourses” (McAllister & Taylor, 2015, p. 86). Rightfully so, this rhetoric has now come under closer scrutiny with several studies examining the partnerships’ “politics of co-production” (Turnhout et al., 2020, p. 15). For example, whereas Dandy et al.’s (2014) analysis of collaborative natural resource management has demonstrated the limited capacity for power sharing within such partnerships due to pre-defined agendas and pre-existing power relations, Turnhout et al.’s (2020) literature review on the subject has highlighted the role of de-politicization dynamics that can actually prevent wider societal transformation taking place. In a similar vein—but put even more provocatively—the performativity of partnership working has been called into question as it could also be seen as simulative politics which may help to sustain the unsustainable status quo (Blühdorn & Deflorian, 2019).

While I agree that more critical analysis of partnership working is needed, I believe that there remains an untapped potential when it comes to the explanatory power of the co-production term itself. Within the literature cited thus far, co-production is usually understood as “a shorthand for participatory modes of knowledge production” (Turnhout et al., 2020, p. 15), and thus remains explicitly dissociated from the analytical conceptualization of this term as proposed by Sheila Jasanoff (2004) and used in Science and Technology Studies. Jasanoff’s “idiom of co-production” (Jasanoff, 2004, p. 1) urges us to look for more accurate and nuanced accounts of “the complicated interplay of the cognitive, the institutional, the material and the normative dimensions of society” (Jasanoff, 2004, p. 17), without ascribing determinism to either social or natural order. To do so, we are directed to the instruments that sustain or establish moral, metaphysical, political or symbolic order, namely all those instances in which identities, institutions, discourses and representations are made (see Table 1).

**Table 1:** The four instruments of co-production (Source: paraphrased from Jasanoff, 2004, pp.39–41)

Instrument	Description
Making identities	the ways in which human, non-human, individual and collective identities are formed that determine order and define roles, with implications for power and knowledge
Making institutions	the setting of common rules, norms, processes, methods, etc. that form the repertoire of socially and politically accepted behaviour that qualifies certain ways of knowing and action
Making discourses	the formation of a common language allowing its users to speak about and act upon a new or old problem in an authoritative way
Making representations	the means and practices used to produce intelligible scientific, social or political representations

As such these four instruments of co-production offer analytical pathways that allow us “to explore how knowledge-making is incorporated into practices of state-making, or of governance more broadly, and, in reverse, how practices of governance influence the making and use of knowledge” (Jasanoff, 2004, p. 3).

Within the field of agri-food studies this has led to important insights, for example, about productivism in agriculture and food systems. According to Iles et al. (2017, p. 965), “the making of productivist regimes is founded on 150 years of the co-production of knowledges, technologies, institutions, cultures, organisms, humans, and markets,” which helps to explain its persistent legitimacy and credibility. By following Iles and colleagues’ suggestion, I will use ‘“co-production” as a storytelling idiom’ (2017, p. 947) and apply it in this paper to partnership working and the specific but widespread problem context of governing agricultural diffuse pollution. Doing so promises multi-dimensional insights into this problem context and into the aforementioned set of instruments.

Identities, institutions, discourses, and representations are of course already seen as important leverage points to address agri-environmental problems, but most studies continue to treat them in isolation. For instance, Inman et al.’s study concluded that “the reality of the situation […] is that a significant shift in identities and behavioural beliefs within farming communities is likely to be required before DWPA [diffuse water pollution from agriculture] mitigation behavior becomes embedded” (Inman et al., 2018, p. 22). The focus on farmers’ identities is a common threat in the literature leading to similar diagnoses (see also McGuire et al., 2013), and yet our understanding as to how institutions, discourses and representations come into play with identities remains rather limited. The same is true for how different governance approaches such as partnership working contribute to identity shifts on and beyond the farm level.

Indeed, the idiom of co-production serves as a reminder to pay attention to shifts within any of the other instruments, too: We know, for example, that laws and regulations alongside social norms and voluntary actions all affect farmers’ behaviour in relation to diffuse pollution (Blackstock et al., 2010), yet we do not know much about how partnership working in turn shapes such formal and informal institutions and what role the other instruments have in re-shaping the rules of the game. Similarly, discourses play an integral part in knowledge and learning processes both on the farm-level (Goulet, 2013) and of course also among all those who seek to govern the land-based sector. Yet again, empirical insights about the narratives, stories and languages used within partnerships to speak about and act upon a shared problem are largely missing. Discourses are however likely to have a significant impact on peoples’ understanding of and action towards diffuse pollution. Lastly, the means and practices used by partnerships to produce intelligible representations of the problem and of the solutions at hand should give us some indication of the politics, rationales and strategies employed by partnerships to bring about desired change. By exploring all four analytical pathways of co-production we should obtain a fuller picture of partnership working and thus gain a better understanding of how this plays out in the local context of Herefordshire.

## A note on methods: studying partnership working through the ethnographer’s lens

To shed more light on partnership working in the case of Farm Herefordshire this paper draws on empirical material gathered through ethnographic fieldwork. Participant observation (Bernard, 2006; DeWalt et al., 1998) has played a pivotal role within this qualitative research approach as it has not only contributed valuable first-hand insights, but also allowed for continuous analysis through regular reiteration and triangulation of data received from other research activities (Patton, 1990) (see Table 2 for an overview). This is noteworthy, because participant observation has rarely been taken into account as a research method when studying agri-environmental collaboration or partnership working (although see Dandy et al., 2014). Most studies have drawn on questionnaire data, interviews, or focus group discussions, even when an explicit “insider” perspective’ (Prager, 2015a, p. 383) or “bottom-up approach” was pursued (Leventon et al., 2017; Prager, 2015a).

**Table 2:** Overview of research methods and data

*Research method*	Participant observation	Semi-structured interviews	Document analysis
*Description of data sources*	Farm Herefordshire steering group meetings On-farm demonstration events Wye catchment partnership meetings Job shadowing / Team meetings Other information events	NGO staff Civil servants Farm advisors Farmers	Reports Leaflets/Brochures Websites Newsletters Videos E-mails Etc
*Total*	n = 30	n = 19	n ~ 70

My own positioning as “participating observer” (Bernard, 2006, p. 347) became possible through long-term fieldwork lasting from October 2016 until December 2017. During this time, I was loosely affiliated as a PhD researcher with one of the organisations involved in the partnership. This charitable organisation was identified during an earlier scoping study^1^ (March–May 2016) and opened the door to the governance network that had developed around Farm Herefordshire. In particular, I was allowed to join staff meetings of a farm adviser team, I was able to job shadow individual staff members of various partner organisations; and I was invited to a number of partnership meetings including those of the Steering Group of Farm Herefordshire. The latter provided a space for the partners to discuss practical and strategic matters of concern and to find consensus about them. The Steering Group is made up of one or two representatives of each partner organization. Joint meetings took place every three months and were held in turns in one of the partners’ offices. Each meeting lasted for about two hours and their nature was characterized by constructive exchange, which gave all participants in principle an equal opportunity to express their opinions, to think out loud about new ideas, but also to wander from one topic to another—on and off the agenda.

While this provided valuable insights into the internals and politics of partnership working, on-farm demonstration events gave me opportunities to observe how the partners engage with farmers. Thus, over the course of this research I also joined several organized farm walks during which farmers and other land-based actors were made aware about the phosphate issue within the Wye catchment. Such events took place across the county and covered a wide range of topics including “Healthy Livestock from Healthy Soils,” “Cover Crops and Conservation Seed Mixtures,” or “Boosting Yield and Profit through Strip Tillage.”

In parallel to participant observation, I conducted 19 semi-structured interviews covering a cross section of organizations and actors who were either directly involved in Farm Herefordshire or in one way or another connected to it (e.g., key farmers). Most interviews were conducted at the workplace or home of the respective interviewee—and in the case of farmers, they were usually accompanied by a transect walk of the farm. All interviews were tape recorded and later transcribed. Each interview lasted for about 1–2.5 h and mainly contained open-ended questions to gather the interviewees’ experiences, opinions, and expectations of partnership working. However, they also served as an opportunity to clarify some of the aspects emerging during the various work meetings I had attended and often provided an atmosphere in which more personal opinions and sensitive issues could be raised and expressed. Providing anonymity for my interview partners in such a tight-knit network was challenging but hugely important due to the sensitive nature of many aspects involved in partnership working and due to the variety of personal viewpoints that often must step back behind wider organisational agendas. I have therefore anonymised all interviewee’s responses (i.e., FH member) and use pseudonyms throughout this text to protect people’s identity.

Lastly, an extensive review of secondary sources related to Farm Herefordshire has provided complementary insights as well as important leads as to how the partnership and the actors involved presented themselves externally. These documents and information sources included Farm Herefordshire leaflets and brochures, partnership reports, emails and newsletters advertising on-farm events, websites of partner organisations, and jointly produced YouTube videos.

## The turn towards partnership working in Herefordshire

### The background story

Despite the increased interest in partnership working it is not a new phenomenon. The turn towards partnership working in the county of Herefordshire has happened gradually and appears to be the consequence of deepening inter-professional relations, as much as it is an attempt to counter further fragmentation in a governance landscape that has become much more diverse since the launch of the first agri-environment schemes in the 1980s (Dwyer, 2014). This becomes obvious when we look at the development of the cross-organizational partnership Farm Herefordshire, which offers farmers guidance and advice on land management under the slogan of “profitable farming, healthy soils and clean water” (Farm Herefordshire, n.d.). Viewed in isolation this partnership could be seen as yet another governance project (Munck af Rosenschöld & Wolf, 2017), which tries to tackle the problem of agricultural diffuse pollution by forming a coalition between the third sector, industry organisations, government bodies and an institute of higher education. Since its official launch on 25 November 2015 it has grown to 12 partner organizations which are listed and briefly described in Table 3.

**Table 3:**
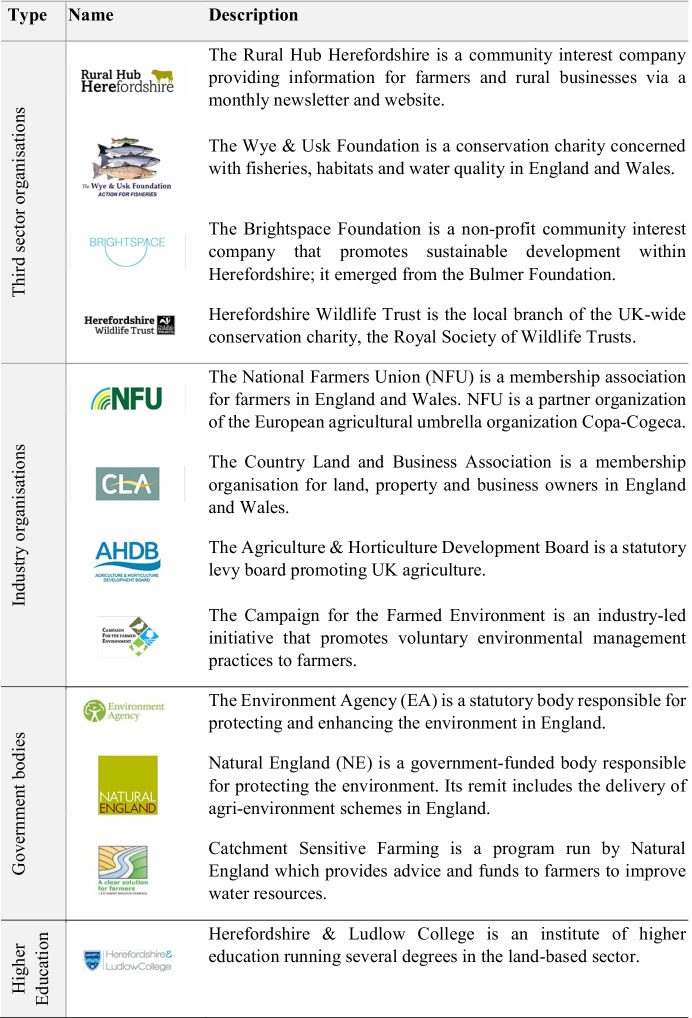
The organizations currently involved in the Farm Herefordshire partnership

However, this coalition did not start from scratch and neither did the commitment to collaborate on the issue of agricultural diffuse pollution emerge suddenly. Many of the organizations that are currently involved in Farm Herefordshire had already been working together for several years trying to promote more sustainable land management practices and agri-environment schemes to farmers. Natural England (NE), the non-departmental government body in charge of protecting and improving the natural environment in England, for instance, employed Catchment Sensitive Farming officers to help reduce agricultural water pollution.^2^ The local liaison group of the industry-led Campaign for the Farmed Environment (CFE) recognized water and soil management as a target area “to demonstrate how the industry collectively takes responsibility for achieving environmental benefit alongside profitable farming” (CFE, 2013), and environmental charity groups such as the Wye & Usk Foundation or the local Wildlife Trust had already worked with farmers, regulators, and the agro-industry on a project-by-project basis.

Overall, these past experiences have helped to establish trust and routinized workflows between those organizations, which allowed them to further their own agendas by working together. For example, by signposting farmers to environmental advice and public monies everyone could gain, either in terms of farmers’ uptake of recommended conservation measures (of particular interest to environmental charity groups), more successful policy implementation as measured in land covered under countryside stewardship agreements (of particular interest to government), or in the form of financial compensations for environmental measures (of particular interest to farmers).

The launch of first formal joint initiatives thus only seemed logical. One of these was Green Futures which already began in 2005. This earlier initiative adopted “a “one stop” and non threatening approach to knowledge-transfer” (Bulmer Foundation, 2015, p. 61), which in practice meant co-organizing and co-hosting regular events to inform the local farming community together about any relevant changes in agricultural policy and environmental legislation. Accordingly, a tight network of key actors has emerged over time and across the traditional governance divides which is keen to engage with all of the 2120 registered farm businesses within the county including those farmers who the members of this network consider as “the hard to reach” (ibid.).

To some extent those efforts paid off as between 60–75 percent of all farmers in Herefordshire make use of an external advisor when applying for agri-environmental funding based on unrecorded, local estimates (FH member, 18. November 2016). While this may help to explain why farmer participation in such schemes lies slightly above the national average (FH member, 2. March 2017), it certainly demonstrates that partnership working in Herefordshire has “a long history” (Wye Catchment Partnership, 2017, p. 3) and was not imposed by any superior entity.

### The common challenge

**Fig. 1:**
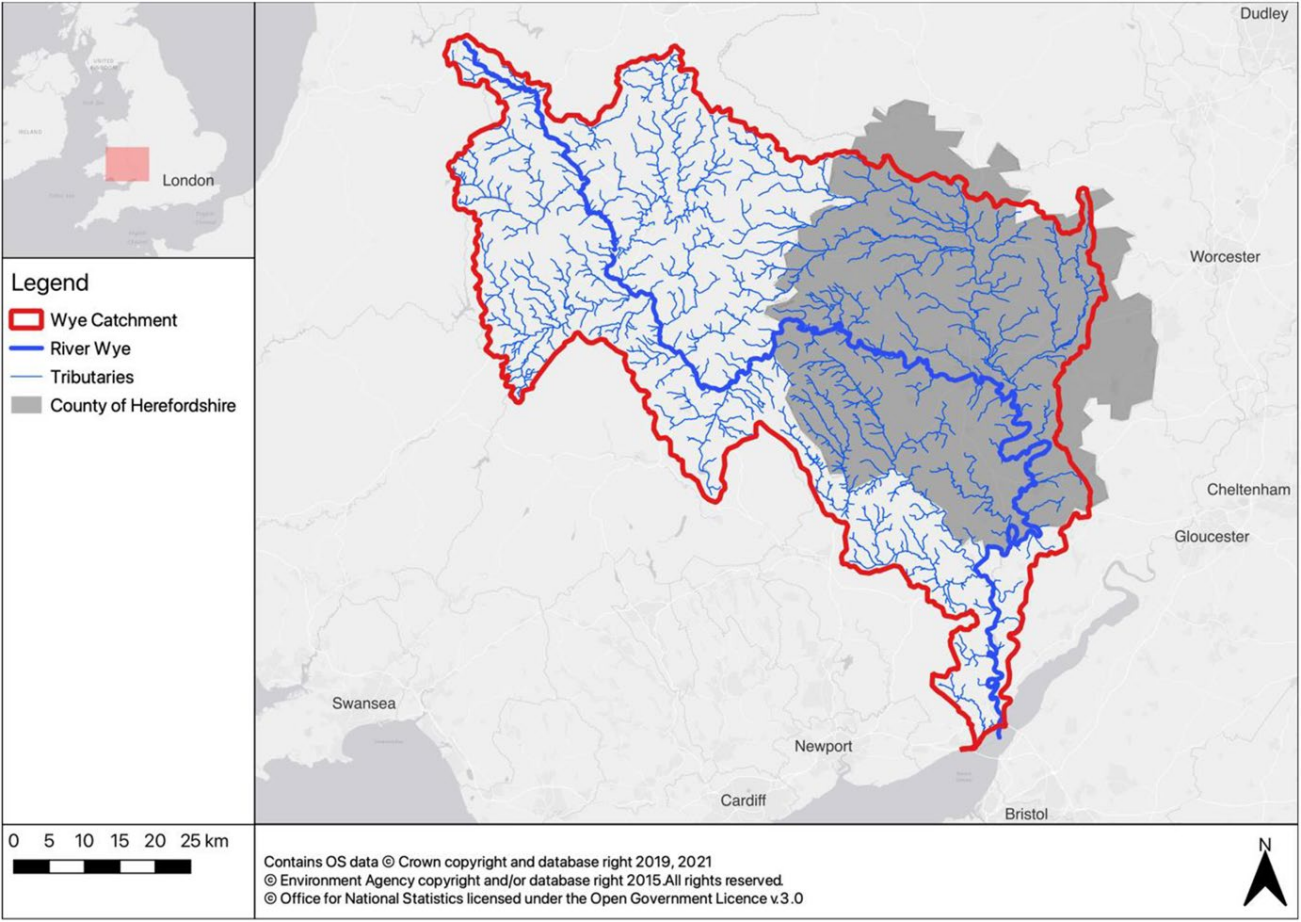
Location of the river Wye, the catchment area and the county of Herefordshire (Realisation: Author)

A whole new dynamic came into this collaborative network when the rivers Wye and Lugg failed to meet their phosphate targets (see Fig. 1 for an overview map). By 2015, 44 out of 50 water bodies in the county were considered to be in moderate, poor or bad status due to elevated nutrient and/or sediment loadings negatively impacting on the water quality and the number and diversity of invertebrates and fish populations. The Wye and the Lugg are home to rare species including otter and Atlantic salmon and hence include stretches designated as Sites of Special Scientific Interest (protected under UK law) and Special Areas of Conservation (protected under the EU’s Habitat’s Directive). The Environment Agency and Natural England declared phosphate losses from farmlands as key pollutants (alongside equal contributions from point source pollution from wastewater treatment plants), which not only adversely affect the environment, but also further housing and industry developments within the county.

Thus, economic growth has come under threat due to a potential development moratorium if the water quality would not be brought back into compliance with environmental regulations. Ultimately this put the future of the county into jeopardy as an aging population, few industries, and low tax revenues seem incompatible with future austerity plans that expect counties across the UK to become less dependent on central government funding. Economic growth and environmental governance have thus become issues of political survival as well as matters of fierce contestations, as ironically one of the few growth factors lies in the booming poultry sector (Davies, 2017), which adds to the diffuse pollution problem through the vast amounts of phosphate-loaded chicken muck readily available to be spread on local farmlands.

Providing advice on better land management has thus become ever more important and eventually led the Environment Agency—in their own words—to “hijack” (FH member, 6 July 2017) the existing collaborative network and use it for the delivery of a Nutrient Management Plan which it had imposed onto the county. The successive institutionalization of partnership working through Farm Herefordshire therefore became politically desired and financially supported, even though neither of the partners actually had a “particular desire to set up another organisation” (FH member, 15 November 2016). This was not because any of the partners was opposed to further collaboration or to the participation of additional government bodies, but because they saw the main problem in the organizational fragmentation of advice to the farming community.

…[F]armers here are faced with an absolute plethora of initiatives and sort of legal requirements and they were clearly getting submerged by all of this. And the last thing we wanted to do was to launch another initiative. So Farm Herefordshire was not intended to be a separate organisation. It was a way of coordinating what we all did and it didn't really matter which of us carried the messages as long as the messages were being carried (Ibid).

This fragmentation of advice in the UK had traditionally been associated with the privatization of public extension services (Curry & Winter, 2000; Klerkx & Proctor, 2013), which appears to have introduced unprecedented levels of complexity for farmers as well as for those who seek to govern. The “plethora of initiatives,” for example has not only become overwhelming, but also contradictory at times, as different actors were giving differing advice to farmers. This is particularly problematic in the context of nutrient recommendations and most obvious in the case of the Nutrient Management Guide RB209 of the Agriculture & Horticulture Development Board (AHDB). This document has for a long time recommended much higher phosphate application rates than what many agronomists, conservationists and farmers felt necessary based on their experience and actual plant-nutrient requirements. Through the formation of Farm Herefordshire and the enrolment of key actors such as the AHDB, the partnership seeks to avoid such contradictions and to create order in an increasingly diverse agri-environmental governance context. As such, Farm Herefordshire represents a collective approach that aims to orchestrate the partners’ individual advice “to ensure consistent messaging” on the phosphate problem (Farm Herefordshire, n.d.). This issue represents a common concern and determined the initial remit of Farm Herefordshire, which is directed at instigating behavioural change among farmers. While this ultimate objective, as well as the involvement of additional government bodies, may be understood as having added a top-down twist to the nature of the partnership, such simplifications based on binary distinctions (i.e., top-down vs. bottom-up) add little to our understanding about the genesis of partnership working in this case. In fact, Farm Herefordshire is a perfect example of the interrelations between such binaries, which have contributed to its ongoing and open-ended development. This is furthermore evidenced by the fact that Farm Herefordshire has become subsumed under the Wye Catchment Partnership, which stretches across various administrative boundaries and which has brought several dozen additional institutional and individual members together “with a shared common goal of protecting and enhancing our rivers, landscape, habitats and wildlife” (see: http://wyecatchment.org). The work of Farm Herefordshire must therefore also be seen in relation to this wider partnership, in the sense that negotiations and interactions in both fora constantly inform each other.

## Exploring the co-products of Farm Herefordshire

The background story in Sect. 4 revealed the politicisation of the nutrient pollution problem which has led to a formalized mode of collaborative action in the county of Herefordshire. Yet, what does this formalized partnership work look like in actual practice? How do the partners of Farm Herefordshire manage to align their agendas and orchestrate their actions? How do they ensure that their collective messages on the phosphate issue are being heard? In this section, I will turn to these questions by attending to the four instruments of co-production set out in Sect. 2. By exploring the everyday practices of partnership working we will be able to see more clearly how identities, institutions, discourses and representations are jointly being made. Although the subsequent analysis is tackling one instrument at a time, it should be noted that their nature is of course interrelated and overlapping.

### Building a collective identity: the premises of neutrality and unity

Despite collegial exchange and genuine commitment to collaborate on the phosphate issue balancing different opinions and organizational politics became critical from the very beginning when the partners had to agree on what the partnership was actually meant to do. This collective identity building was a lengthy process involving many careful decisions. For example, the nomination of the director of the Brightspace Foundation to act as the chairperson of the partnership was one of these decisions as he recalled:

There were a lot of politics involved in that. And one of the reasons why we chair Farm Herefordshire is that we are in a way the honest broker in the midst. We do work with farmers…, but we tend to work primarily with orchard owners. So we have a track record in the land-based economy, but we were not a membership organisation and we're not there to promote a particular issue like fishing,… or enforcement …so we ended up being the sort of gate keeper and the partners agreed that we would be the accountable body for any funding. (FH member, 15 November 2016).

As a non-profit organization, the Brightspace Foundation once belonged to the well-known Hereford-based cider maker Bulmers, but nowadays operates independently and broadly advocates sustainable development within the county through collaboration, open data and local solutions. Given its “non-conflicting” background all other partners saw it best positioned to help relieve any political tensions that were likely to arise by trying to gather different interests and personalities around the same table.

**Fig. 2:**
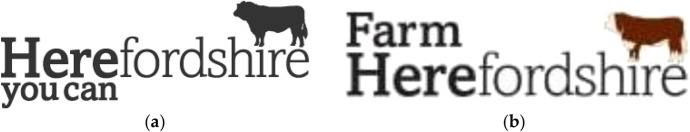
(**a**) Place branding logo of Herefordshire county (Source: https://www.hereyoucan.co.uk); (**b**) the modified logo of Farm Herefordshire (Source: https://www.wyeuskfoundation.org/farm-herefordshire)

An equally careful decision was made about the branding of the partnership. For the choice of the logo the partners considered it important to demonstrate that “Farm Herefordshire has no baggage” (FH member, 6 July 2017). The logo was inspired by the place branding used by local county authorities including the tourism and business board (Fig. 2a). The Herefordshire master logo contains the iconic Hereford bull and with its tagline “Herefordshire you can” (emphasis in original) plays on the famous slogan used by the Obama campaign team in the 2008 US presidential elections.

Slightly re-arranged to reflect the desired neutrality of “Farm Herefordshire” (emphasis in original) the logo neither directly discloses any of the involved partners, nor does it allude to the negative phosphate problem (Fig. 2b). Instead, it conveys the impression of a collective identity around a common place of farming and belonging by drawing on the agricultural heritage within the county. Thus, the members of Farm Herefordshire felt it was important to camouflage the individual organization’s interests and reputations by choosing a logo that was perceived to be less biased than say, for instance, the fish or badger depicted on the logos of the environmental charity groups (see Table 3 in Sect. 4.1). On one side this is based on the realization that each partner’s ability to engage with farmers—and therefore to contribute to the overall success of Farm Herefordshire—remains constrained by their own reputations and capacities. On the other side this also demonstrates a growing recognition among the partners that complex agri-environmental problems such as diffuse pollution can only be solved by loosening the straitjackets of existing organizations. Accordingly, all partners regularly emphasized in the steering group meetings—as well as in the Wye Catchment Partnership meetings – the need for sharing information and data and for de-siloing knowledge in order.

[to get] everybody to work together, to bring their resource to the table, be it money or skill or what have you and to leave all the badges at the door […]. Farm Herefordshire is that badge and we work underneath it. (FH member, 30 January 2017)

This implies that identities also need—and indeed begin—to change on the organizational level of the involved partners and not only on the farm level itself (Inman et al., 2018). This commitment affords that one-sided interests necessarily have to step back behind the shared objective, which is obviously not easily achieved. However, in return this unity holds the potential to bridge the fragmentation of mitigation advice and helps to strengthen “the credibility of the message content” (Blackstock et al., 2010, p. 5635), which remains a major challenge within a sector that tends to downplay its negative environmental impact in general (Silvasti, 2003a), and particularly with respect to water resources (Blackstock et al., 2010).

Thus, what might appear at first as a potential de-politicisation of the problem matter, is actually far from it. The premises of neutrality and unity rather constitute a pragmatic attempt to make headway in a field that has traditionally been much more divided and resistant to change—not least due to competing demands and interests.

### Agreeing on joint institutions: the role of agenda-setters and the testing of boundaries

**Fig. 3:**
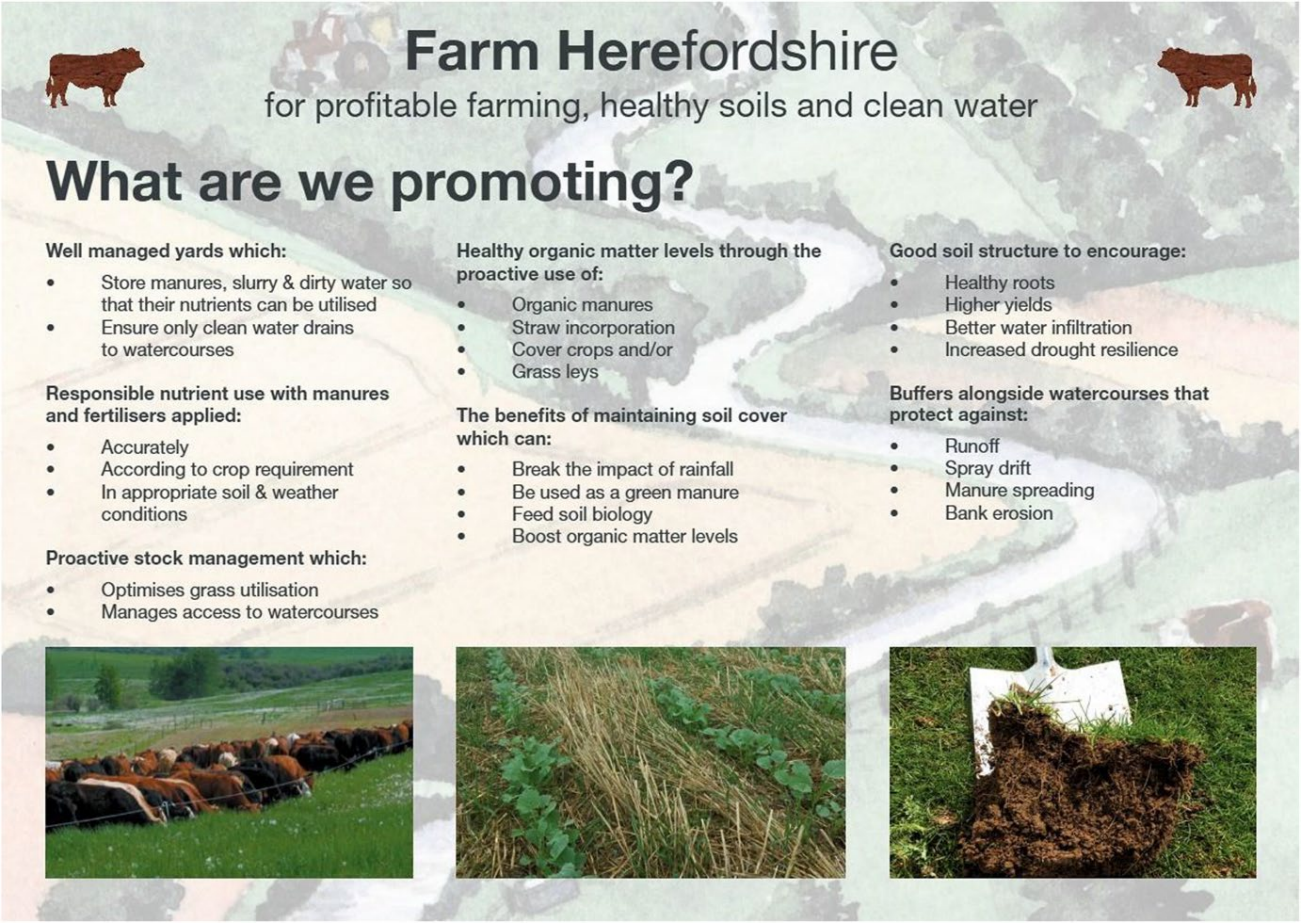
The specific farming practices promoted by Farm Herefordshire (Source: Farm Herefordshire leaflet)

In practice this new organizational demand means “a lot of time talking about things that appear to be quite (…hm…) miniscule like the wording on the leaflet” (FH member, 15 November 2016), which contains the diffuse pollution mitigation measures that Farm Herefordshire promotes to farmers (Fig. 3). In this context the influential role of agenda setters, who have the capacity to steer the partnership in specific directions, becomes apparent. Here, one representative of an environmental charity group stands out who not only acts as a knowledge broker, but also as a diplomat who is capable of traversing different working cultures, mediating confrontational situations, and translating new scientific insights into more colloquial parlance. This key person, called Ella,^3^ was raised on a farm, had previously worked for a governmental partner organization, and had recently conducted a farming-industry sponsored study on re-engaging farmers with their soils. On the one hand such people skills are appreciated by the other partners for they help to overcome inter-organizational tensions that previously existed:

The [name of member organization] has been a very, very interesting scenario, cause you know there's quite strong characters there and …erm..[a specific activity] is obviously their top game, which doesn't actually often lie necessarily with, you know, some of the other side of things, but…Ella's brilliant, she is absolutely fantastic, so I think she's made a huge difference, you know to that organisation,… trying to get rid of the tensions, (FH member, 30 January 2017)

Ella’s personal interest in soil health has also been instrumental in bringing this topic to the partnership table and in aligning it with the phosphate problem. With at least three out of the seven mitigation measures directly related to soil health (i.e. ‘soil cover maintained’; ‘good soil structure’; ‘healthy organic matter levels’), it has in fact become institutionalized within the specific land management practices promoted by the partnership.

More controversial than the unifying topic of soil remained the question whether or not all livestock should be fenced off from watercourses to prevent riverbank erosion and water contamination from livestock poaching^4^ and defecation. This other “best practice” was demanded by several partners but opposed by a farming industry representative, which explains the vague formulation of “proactive stock management.” Examples like this show that the outcomes of the steering group discussions are always compromises that reflect the partners’ standpoints gained through their specific work experiences, commitments and convictions, but also their willingness to make concessions. After all, fencing-off livestock from watercourses is not off the agenda. It has been carefully rephrased and left to the discretion of each partner to interpret its meaning. Thus, leaving all the badges at the door does not eliminate different viewpoints, but the steering group certainly provides a space for more open discussions that allow the testing of boundaries.

The testing of boundaries becomes even more apparent with the partnership’s maturation and its discussions moving beyond the original remit of phosphate reductions. For example, over the course of this research further common ground was explored on the use of glyphosate, the importance of biodiversity and the role of the agri-food industry. The first topic was raised by the chairperson of the partnership with the intention to provide farmers with a joint recommendation about alternatives shortly before the EU decided to prolong the license for this herbicide. While this suggestion was welcomed in principle, both the National Farmers Union and the Environment Agency representatives were unable to commit to this idea due to a national NFU campaign running in parallel in support of glyphosate, and on the grounds of political neutrality on part of the EA, respectively. Full support on the other hand received the idea to retain the advice on biodiversity provided by the Campaign for the Farmed Environment, who was confronted with budget cuts. This decision allowed the group to demonstrate their solidarity with another partner organization and showed their appreciation to act upon issues that are related to diffuse pollution. Wider engagement with the agri-food industry on the other hand came into play as a potential solution to secure core-funding for the partnership itself. Although the EA representatives had provided initial start-up funding for Farm Herefordshire by juggling different pots of money around, they regularly made clear that a long-term solution was needed. Thus, bringing in corporate money was discussed on several occasions as well as the idea to register a trademark for Farm Herefordshire that could potentially culminate in a marketable food label. Such insights show the organic and open-ended nature of Farm Herefordshire. Despite pre-defined agendas that everyone brings to the table there seems to be room for further explorations, which may as well add to the future scope of the partnership and the repertoire of jointly agreed institutions.

### Making representations with the help of the “good guys”

In conjunction with the making of a collective identity and shared institutions we can observe the co-production of representations. This is most obvious on the farm level where the partnership’s messages and joint actions try to redefine what constitutes good agri-environmental practice within the county. For this the protagonists of Farm Herefordshire actively link and augment the cultural concept of the good farmer (Burton, 2004; Silvasti, 2003b) with the agri-environmental measures and technologies that have been recognized by the partners as appropriate to redress the phosphate problem.

Farm walks—the first pillar in the engagement strategy of the partnership—are vital in this context because they allow the partners to demonstrate how good practice looks in real-life settings (see also Blackstock et al., 2010).^5^ These free and public events are either organized separately as Farm Herefordshire farm walks or “piggy-backed” onto one of the partners’ otherwise planned on-farm activities. Most importantly they are hosted on farms which are considered by the partners as examples of “best practice” within the county. The protagonists are recruited through the partners’ own network, with most of them belonging to a loosely organized group of farmers who already share an interest in more sustainable land management. This informal group has already existed before the launch of Farm Herefordshire, but now serves the explicit purpose of coordinating the work of the partnership with the local farming community. Joint suppers with this group of farmers were organized twice a year during which the members of Farm Herefordshire sought feedback and legitimacy for their messages and future course of action.

The farm walks as such seek to combine a farmer-led approach to knowledge transfer with expert-advice from the involved organizations. Some observations from one of these events help illustrate their character: For example, a well-attended meeting took place near Kington on Tuesday the 24^th^ of January 2017 attracting more than 50 participants including farmers, farm advisers, and local residents. Following the usual procedure, the Rural Hub Herefordshire had advertised the event in its monthly newsletter which reaches more than 1700 recipients. The event was hosted by the Evans family, who run a mixed farm that is partly certified organic, and who had previously sought environmental advice from the Wye & Usk Foundation. After complimentary tea/coffee and a short welcome speech by the farmer and his son, two facilitators—one from the Wye & Usk Foundation and one from NE—took over and invited the audience to join them on a farm walk to “find out how good soil management can help to boost profitability and health of your cattle and sheep” (Farm Herefordshire event leaflet n.d.). The walk lasted for about two hours and included several thematic stops during which the two facilitators actively tried to engage the audience in discussions around the Evans family’s farming practices. This covered their accurate application of fertilizer and use of soil testing, their careful management of soil structure and biology for profitable grassland, their strategy to maximize the quality and quantity of forage production, as well as their responsible wormer use for healthy soil invertebrates and mineral balance in livestock.^6^

**Fig. 4:**
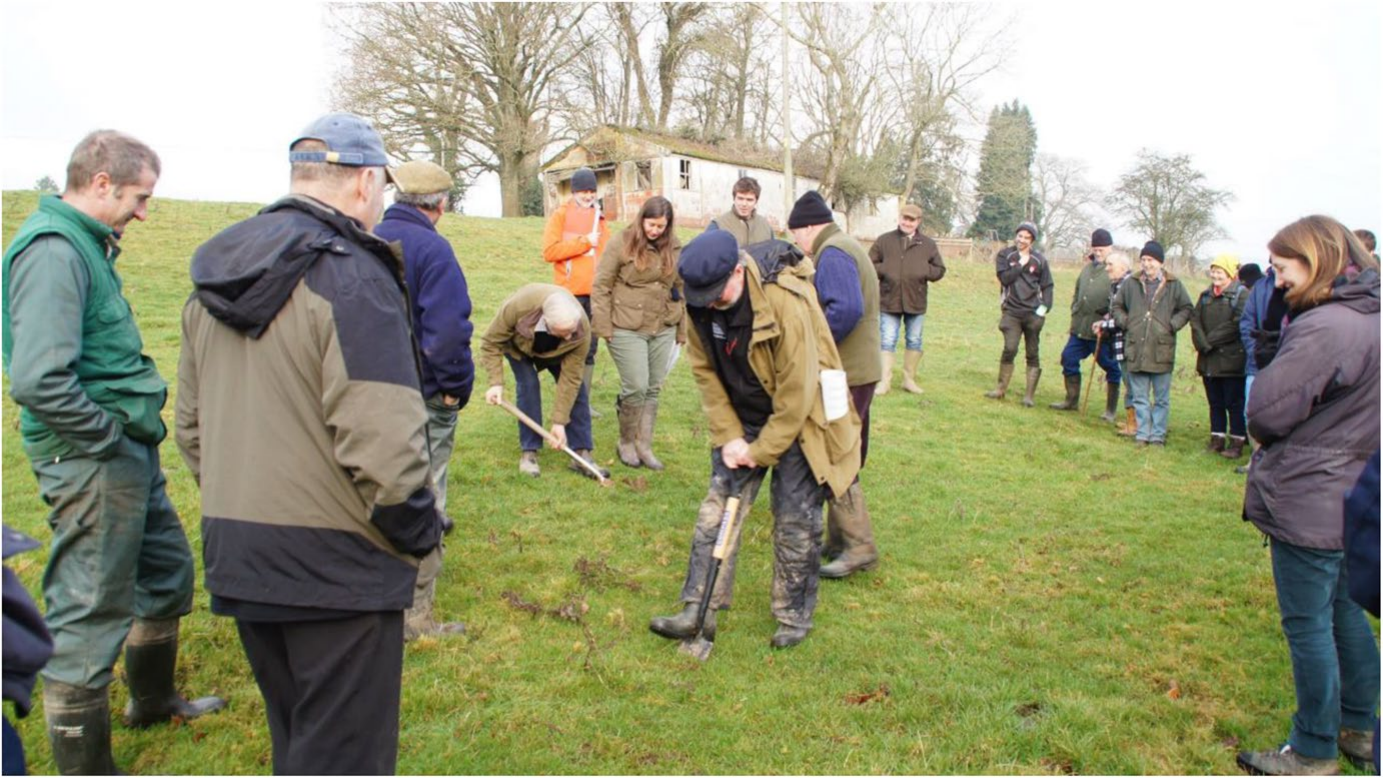
Participants take soil samples to assess the health of soils during a farm walk organized by Farm Herefordshire (Source: Author)

To support and validate the benefits of the host’s good land management practices the facilitators stepped in and drew on some of the earlier mentioned people skills to mediate and further inform the discussions (e.g. by explaining scientific terms and new insights in “farmer speak”). Moreover, they also relied on a sensual engagement with the problems and solutions at hand. Thus, taking a spade, digging a hole, feeling the soil structure, and counting the worms were integral parts of each of these farm walks (Fig. 4).

This was done to re-engage farmers with their soil, to strengthen their attentiveness and appreciation for soil organisms and functions (Krzywoszynska, 2019), and to convince them of the need to conduct regular soil tests to avoid over-fertilization and by association further phosphate pollution. This emphasis on a direct engagement with soil is noteworthy because tactile encounters are important for knowledge about more sustainable farming practices to become embodied (e.g. Carolan, 2007, 2008, 2011). In fact, as Vankeerberghen and Stassart (2016, p. 397) suggest hands-on engagement like this “leads to a cognitive detachment from seeing the soil as a “substrate” to seeing it as “living soil.””.

This attempt to re-make what it means to be a good farmer is noteworthy for two reasons: First, it shows that its defining values and principles are never exclusively negotiated between farmers, but within wider networks of actors, which reflect the changing interest in and expectations of agriculture (see also Burton et al., 2020). Importantly, non-farming actors—such as in this case government agencies, environmental charity groups or industry bodies—equally subscribe to re-negotiated versions of a good farmer in order to accommodate their range of (changing) values with the farming sector. Second, it clearly constitutes an attempt to construct regional or place-based versions of the good farmer by creating attachment and responsibility to familiar and (under)valued landscape features such as the river Wye or farmers’ own soils. As such this supports the “emerging recognition that subjectivities associated with the “good farmer” concept are subject to spatial, contextual and temporal shifts and contestations” (Wheeler et al., 2018, p. 667).

### Reconciling contested discourses: the promise of “Eco-productivism”

What all the “good farmers,” who collaborate with Farm Herefordshire, have in common is that they are very well connected to one or more of the partner organisations. They are early adopters of the promoted land management practices. They are keen to host and showcase their business and farming practices, and they are confident to speak in front of a live audience or a video camera, no matter if their background is in mixed farming, arable, organic, conventional, no-till, large-scale estate or smaller-scale family farming. This range of good farmer’s profiles is useful as it helps the partners to target and speak to the wide spectrum of farming within the county. “The good guys [who] are doing it anyway” (Wheeler et al., 2018) therefore also play a key role in the best practice videos,^7^ which form the second pillar of Farm Herefordshire’s engagement strategy. Each of these videos is dedicated to one of the aforementioned good agri-environmental practices and follows a similar storyline. The scenes capture an interview situation between a Farm Herefordshire representative and a selected farmer who talks about his personal experiences with one of the promoted practices.

**Fig. 5:**
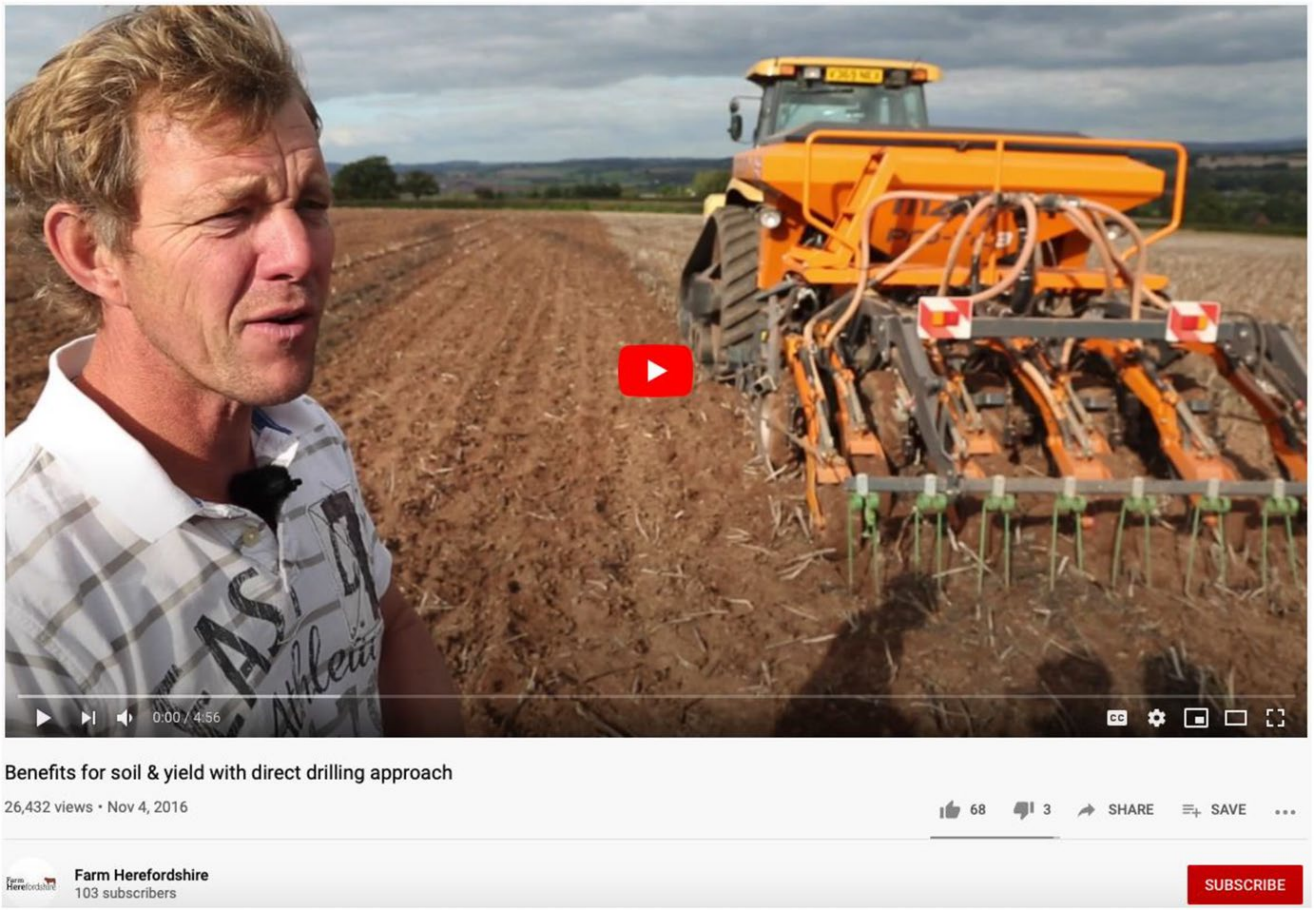
Screenshot of Farm Herefordshire YouTube video, showing John in front of his Mizuri strip-till drill (Source: https://youtu.be/XBdruGJzkYA)

In the most frequently watched video on YouTube,^8^ John Joseph, an arable farmer from South Herefordshire, talks about the benefits of conservation tillage for soil health and yields (Fig. 5). According to John, the soils on his farm were “farmed out” when he took over the land, and he even once had “watched the soils washed down the village all summer long” when he rented out his land to a potato grower. What stands out in his video statements is that even though other farmers mismanaged John’s soils he assumes the collective responsibility for the “damage **we** were doing to the soils with continuous cultivations, mineralization and nitrogen and the release of the carbon” (emphasis by the author), which eventually convinced him to do “something different.” For him this difference lay in “switch[ing] over to the Mizuri system [strip tillage] after the wet year of 2012.” Since then he is “trying not to drive on the land at all other than … when it's essential”—and this new strategy pays off in terms of “savings,” the desired “crumb structure” of the soil, and higher yields contrary to his own expectations as well as those of his peers.

First-hand accounts such as this one show how a differentiated understanding about soil can serve as a tipping point towards conservation agriculture (Vankeerberghen & Stassart, 2016). And moreover, it helps the involved actors to dismantle oppositions between productivity on the one hand and environmental conservation on the other, by emphasizing their synergies and by constructing a narrative around efficiency gains. The emphasis on profitability was a common denominator that interestingly enough was not only important to the farming industry associations, but equally so to the environmental charity groups. The latter have long realized the potential that a narrative around profitability holds as an entry-door to the fields and minds of farmers.^9^ Thus, as much as stories like this demonstrate the fundamental importance of healthy soils for agricultural production, farmers can make savings and improve their business results when they adopt better land management practices and novel technologies. In the end this shall demonstrate that the problem of diffuse pollution can be solved without adversely affecting farmers' incomes; indeed, farmers like John shall show how farming “greener” can keep—or put farmers—in the black.

This constructed reconciliation of previously contested discourses suggests that farmers are no longer expected to uphold different values simultaneously (Emery, 2010, 2014), but to recognize their mutually constitutive character. Thus as in the case of similar collaborative initiatives elsewhere, farmers’ “productivist and conservationist identity standards [are] moving toward congruence” (McGuire et al., 2013, p. 66). I would posit that this congruence signals the much needed turn away from ecological simplification—which has been so fundamental for the rise of productivist agri-food systems (Iles et al., 2017)—towards something that we could call “eco-productivism” (see also Griffon, 2013). Eco-productivism in this context lies somewhere in-between other powerful but potentially dividing concepts and ideas—such as organic vs. conventional, agro-ecology vs. sustainable intensification, land sparing vs. land sharing—and thereby perfectly reflects the non-confrontative and pragmatic nature of working in partnership for better land management practices.

## Conclusion: a hopeful appraisal of partnership working

This article has told the story of Farm Herefordshire—a local partnership initiative that raises awareness about agricultural diffuse pollution on the English side of the river Wye. One of the article’s contributions to the literature lies in the use of ethnographic research methods which has allowed me to look beneath the surface of the partnership approach in agri-environmental governance and to uncover some of its everyday aspects and meanings for those actors who are directly involved. The study has revealed that partnerships like Farm Herefordshire build on a long history of inter-professional relationships that have developed in response to the fragmentation of agri-environmental advice to the farming sector. This common history has created trust among the partner organisations and contributed to better coordination, but it has also shaped, and to some degree pre-determined, the scope, membership structure and ways of more formal partnership working. Studying this phenomenon with some of the organisations involved has also shown that day-to-day partnership activities can be cumbersome and mundane. Building a partnership, agreeing on its scope, its key messages and actions requires a lot of time for meetings, discussions, and consensus building. Diplomacy is key here, but this is fraught with compromises which often reflect the lowest common denominator. Nevertheless, these laborious and long-winded processes which are inherent to partnership working allow agenda-setters to make their mark on the partnership and by doing so test new and potentially more progressive common ground. These mundanities of partnership working rarely enter the limelight of research studies, but as this paper has shown they are essential to the functioning of such initiatives.

The second contribution of this paper lies in utilising Jasanoff’s idiom of co-production which has allowed me to document and account for the less tangible, but very powerful dimensions that Farm Herefordshire is trying to rework. The partners actively nudge farmers towards better land management practices via their attempts to re-shape identities, institutions, representations, and discourses. The nature of any of these four instruments is rigid and difficult to change, which makes the concerted efforts of the partnership noteworthy and critical. Here in this context these concerted efforts involved the partners’ careful framing of the phosphate pollution problem and how they made it appear less political by emphasizing the premises of neutrality and unity. Such efforts involved the negotiations and compromises that the partners had to make about acceptable land management behavior which qualifies certain practices over others. The concerted efforts also became visible in the representations made about good farming in Herefordshire. These representations became more powerful through the enrolment of the “good guys” who have acted as ambassadors and peer-to-peer communicators of good practice and allowed the Farm Herefordshire partnership to appeal to farmer’s self-accountability and a shared sense of place that is worth protecting. Lastly, the partnership’s attempts to change dominant discourses became clear in the formation of a common language which has allowed the partners to speak about and act upon the phosphate problem in an authoritative way. In this common language certain storylines and technologies have become aligned in such a way that tackling diffuse pollution not only seems possible, but actually makes perfect economic sense to any farming business. All of this demonstrates the many frontiers that this partnership is working on simultaneously, as well as the collective effort and hard work that is necessary to create common ground for incremental, but broad-based change.

As such partnerships like Farm Herefordshire are certainly no silver bullet (McAllister & Taylor, 2015) which are able to solve complex agri-environmental problems or fundamentally transform power relations that govern agri-food systems. But at least in this case the analysis clearly demonstrates that the partnership approach does not aim to sustain the status quo as suggested by other commentators (e.g., Blühdorn & Deflorian, 2019). Farm Herefordshire represents a soft, non-confrontative and pragmatic approach to working with the mainstream farming system, and it is exactly here where much of its value lies. The partnership shows a clear ambition to raise awareness about the phosphate issue and about better land management practices by targeting the entire “farming community” of Herefordshire including “the hard to reach.” To that end Farm Herefordshire has been successful as the partners managed to engage with almost 500 farmers during the first two years of the initiative, which represents almost a quarter of the county’s registered farm businesses. The partnership activities have also supported the partner organisations with the delivery of their individual work streams—be that in terms of increasing the length of riverside fencing or concreting farmyards to avoid run-off. Even though it is impossible to clearly attribute the contribution that Farm Herefordshire has had on these activities due to the partner organisations’ multiple work streams and sources of funding, it is these small steps and successes which show that partnership working is not powerless, but indeed able to contribute to changing social-ecological orders on the local level and in the material environment.

Whilst this study provided insights into some of the everyday aspects and dynamics of partnerships working, future research studies should consider longitudinal evaluations of partnership working to further establish the direct and contributing factors on engendering behavioural change on the farm level. The level of fragmentation of farmers advice is also likely to differ in other national contexts and so are the reasons and justifications for the use and establishment of partnership working not necessarily the same elsewhere. Therefore, comparative insights from other national contexts could help inform the evidence of partnership working.

## Data Availability

The data of this study is available from the author upon reasonable request.

## Footnotes

1 The scoping study followed the principle of snowball sampling and included ten semi-structured interviews with key informants as well as a number of site visits including to the study area.

2 This programme was recently integrated into the UK’s agri-environment schemes and can now be accessed by farmers through the mid-tier element of the Countryside Stewardship.

3 Pseudonyms are used throughout this paper to protect the identity of all research participants.

4 Livestock poaching “is the name for damage done to grass and the underlying soil when it is trempled by livestock in wet conditions” (AHDB Beef, Lamp, 2016, p. 12).

5 Such on-farm demonstration events have allowed Farm Herefordshire to engage with around 500 farmers during the first 2 years of the initiative.

6 The discussions around the last two topics were facilitated by a local veterinarian who was invited to the event by the organizers. This underlines the partnerships commitment to collaborate with like-minded actors.

7 The main motivation behind these videos was to reach a larger audience, especially those local farmers who would never show up at a physical meeting. It remains questionable though whether or not these farmers would access Youtube videos instead.

8 With more than twenty-six thousand views since November 2016 this is the most frequently watched video on the YouTube channel of Farm Herefordshire.

9 Farm advisers of the Wye & Usk Foundation, in particular, resorted to this narrative when providing free and confidential advice during farm visits. In both face-to-face communication and their final farm reports they emphasized the potential economic benefits to be gained by farmers when they chose to implement various conservation recommendations.
